# Safety Assessment of Glucose-Lowering Drugs and Importance of Structured Education during Ramadan: A Systematic Review and Meta-Analysis

**DOI:** 10.1155/2022/3846253

**Published:** 2022-02-18

**Authors:** Rashmi Shiju, Ayesha Akhil, Smitha Thankachan, Jaakko Tuomilehto, Monira Al Arouj, Abdullah Bennakhi

**Affiliations:** ^1^Office of Regulatory Affairs, Dasman Diabetes Institute, Kuwait; ^2^Department of Public Health, University of Helsinki, Helsinki, Finland; ^3^Public Health Prevention Unit, Finnish Institute for Health and Welfare, Finland; ^4^Diabetes Research Group, King Abdulaziz University, Jeddah, Saudi Arabia; ^5^Clinical Trials Unit, Dasman Diabetes Institute, Kuwait

## Abstract

**Background:**

Ramadan is the sacred month of the Islamic Hijri (lunar) calendar, and during this entire month, healthy adult Muslims abstain from eating and drinking from dawn to sunset. Muslims with Type 2 Diabetes Mellitus (T2DM) who choose to fast during Ramadan encounter major risks such as hypoglycemia, hyperglycemia, diabetic ketoacidosis, dehydration, and thrombosis. Although patients with poor glycemic control and on multiple insulin injections are at high risk and exempt from fasting, many still insist on it. Thus, healthcare professionals play a pivotal role in managing diabetes-related complications in patients who fast during Ramadan. However, there is a lack of standard guidelines to be followed in association with structured education and administration of drugs and dosage. Therefore, we performed a systematic review and meta-analysis of the literature to determine the safety and efficacy of different classes of drugs and the importance of structured education during Ramadan.

**Methods:**

In this review, an extensive PubMed search was performed to obtain literature on T2DM patients who fast during the month of Ramadan until the year 2020. Preference was given to fully downloadable articles. The articles were extracted based on the eligibility criteria. The extracted data were analyzed using Review Manager software version 5.3.

**Results:**

A total of 32 articles were included for the review and 7 studies for meta-analysis. Majority of the studies demonstrated the importance of structured education either as a group session or as a one-on-one session with the healthcare professionals in preventing diabetes-related risks during Ramadan. As far as glucose-lowering drugs are concerned, DPP-4 inhibitor combined with metformin remains the drug of choice for T2DM patients who fast during Ramadan. The newer class of glucose-lowering agents appear to lower the risk of hypoglycemia in comparison with sulphonylureas, while among sulphonylureas gliclazide is relatively safe. The meta-analysis indicates that DPP-4 inhibitors would significantly reduce the risk of hypoglycemia as compared to sulphonylurea (odds ratio = 0.38, 95% CI: 0.26 to 0.55, *p* < 0.00001).

**Conclusion:**

The results of our systematic review show that structured education and counselling by healthcare professionals can be an effective tool in preventing complications associated with fasting during Ramadan in people with T2DM. Additionally, the safest class of oral glucose-lowering drugs preferred during Ramadan fasting in T2DM patients is DPP-4 inhibitors.

## 1. Introduction

Among the religions in the world, Islam accounts for approximately 22% of the world's population. As of 2010, the total number of Muslims was estimated to be over 1.5 billion [1]. Though Muslims are found in all five inhabited continents, more than 60% of the global Muslim population predominantly belong to Asia while about 20% is in the Middle East and North Africa (MENA). In the European continent, Muslims comprise approximately 7% of the overall population [1, 2]. Early Islam emerged in the Arabian Peninsula region which justifies the fact that this region has the highest concentration of Muslims. The total population of MENA region grew drastically from 3 million in 1870 to 8 million in 1950 and to 85 million by 2020 [3].

According to the International Diabetes Federation Atlas 2019, diabetes has emerged as one of the rapidly increasing global health emergencies. By the end of 2019, around 463 million people were predicted to have diabetes in the world. This number is estimated to reach 578 million by the end of 2030 and 700 million by 2045. A similar trend is estimated in the MENA region; a whopping 96% increase in the diabetes population is predicted in this region by 2045 in comparison with the population data of 2019. In the Arab world, the prevalence of Type 2 Diabetes Mellitus (T2DM) in adults (20-79 years) is reported to range from 4% to 22% [4]. A host of factors including dietary and lifestyle changes brought about by rapid economic development, increased urbanization, and the transition to a sedentary lifestyle led to a rise in the prevalence of T2DM [5]. This was also found to be significantly associated with higher Gross Domestic Product (GDP) (*p* = 0.020) and energy consumption in diet (*p* = 0.017) [6].

Muslims follow the Islamic lunar calendar, and Ramadan falls in the ninth month of this calendar [7]. Muslims across the globe observe the holy month of Ramadan with high reverence as it is one of the five pillars of Islam and forms an inherent part of the Muslim faith [1]. During this period, healthy adult Muslims refrain from any form of food or drink orally or parenterally from dawn to dusk for hours ranging from 10 to 20 hours according to geographical location of the region and seasons with an exception to exempt categories such as the sick, travelers, prepubertal children, pregnant, nursing mothers, and women during their menstruation [8–12]. Despite their health constrain, many such people insist and observe fast during this period [13]. Fasting Muslims usually consume two main meals during Ramadan, one before sunrise, known in Arabic as “Suhoor”, and the other after sunset, known as “Iftar” [7, 14].

People with T2DM who intend to fast in Ramadan are encountered with major risks such as hypoglycemia, hyperglycemia, diabetic ketoacidosis, dehydration, and thrombosis due to an elevation in adrenaline, noradrenaline, and cortisol hormones [15, 16]. Throughout the period of fasting, there is a restricted fluid intake which results in higher chances of dehydration and could further deteriorate with a long duration of fasting in hot and humid climatic conditions [15].

Although, globally, a large number of Muslims fast during Ramadan, there is no clear scientific agreement on the guidelines or advice that should be followed by patients who are fasting. The information on the safety and efficacy of glucose-lowering drugs during Ramadan fasting is still scanty [17–19]. The EPIDIAR study was the very first study to provide information on fasting-related issues in diabetic patients during the month of Ramadan. This study consequently led to various management strategies including Ramadan-related education, change of medication with meals, and recommendations for this area in diabetes [8]. The IDF-DAR (International Diabetes Federation-Diabetes and Ramadan) Practical Guidelines provide healthcare professionals with consistent background information and practical recommendations which are aimed at delivering the best possible care and supporting to patients with diabetes during Ramadan, while minimizing the risk of complications [13]. Recently, American Diabetes Association (ADA) and European Association for the study of Diabetes (EASD) published new consensus statement for the management of diabetes [19]. Following this statement, recommendations for management during Ramadan were prepared by an expert group (Ibrahim et al.). The objective of the present paper is to systematically review and investigate the safety of glucose-lowering drugs administered, structured education, and lifestyle changes in people with diabetes during Ramadan fasting.

## 2. Materials and Methods

A thorough electronic search on PubMed was performed from the year 1950 to June 2020. The search is specifically aimed at retrieving publications which reported the effect of various classes of glucose-lowering drugs during Ramadan on health outcomes of people with T2DM. All possible sources were used to download the full-length article. The key words for search were “Fasting” [MeSH Terms] AND “Diabetes Mellitus” [MeSH Terms]. With Ramadan as MeSH Terms, the list of searched items was minimal. Thus, Ramadan articles were sorted out from the fasting list of articles. The title and abstract of the studies were evaluated for eligibility. All studies, in which diabetes mellitus and Ramadan fasting were discussed, were considered and determined for the eligibility as per the inclusion criteria. In studies with mixed patient populations [Type 1 DM (T1DM) and T2DM], data on the T2DM group were included and extracted. Cross-reference from the selected articles was also searched. Finally, a total of 248 full length articles were considered. The numbers of studies included in qualitative and quantitative synthesis (meta-analysis) were 32 and 7, respectively. The design of the systemic review search was based on Population Intervention Comparison Outcome Study Design (PICOS). The population for the review included patients with age greater than or equal to 18 years, male and female with T2DM for more than one year and fasting during Ramadan. Studies on children, gestational diabetes, pregnancy, T1DM, and nondiabetic patients were excluded. The outcome for this systematic review was to determine the effect of glucose-lowering drugs and effect of structured education during Ramadan.

**Figure 1:**
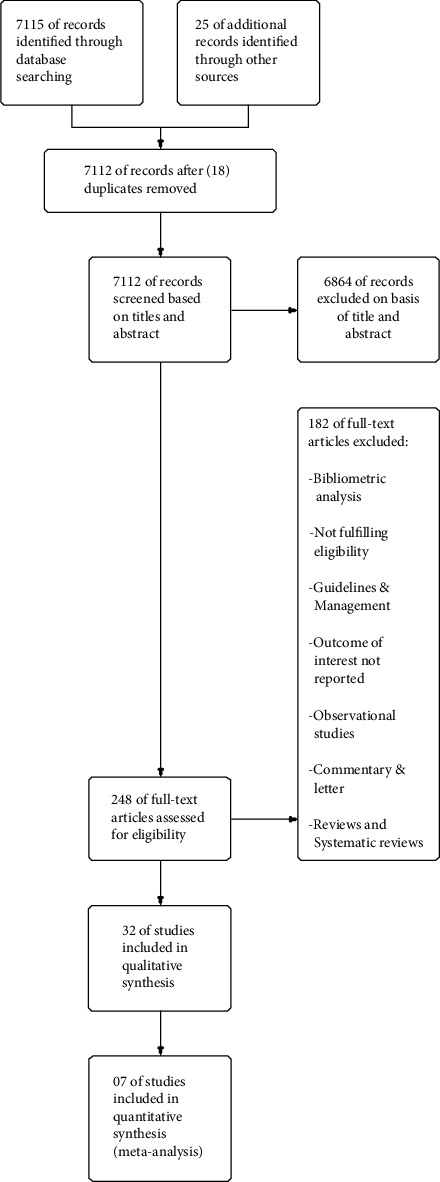
PRISMA flow chart.

The study quality was assessed in accordance with the guidelines for systematic review by Cochrane collaboration. The criteria included adequate sample size, detailed eligibility criteria, description of the population, description of the intervention, control or comparison, outcome assessment, well-defined statistical measurements, accounting for confounders, discussion, and conclusion supported by findings. Data were extracted from the eligible articles using predefined template of data extraction. The PRISMA flow chart (Figure 1) depicts the summary of literature search showcasing the reason for exclusion, characteristics of included studies, participants, interventions, and outcomes.

The End Note X7 software was used to compile and store all the related references. All study designs such as epidemiological studies, observational studies, surveys, prospective studies, cross-sectional studies, randomized control trials, open-label studies, comparative studies, and questionnaires were included in the meta-analysis. Review Manager version 5.3 was used for meta-analysis of DPP-4 inhibitors versus sulphonylurea safety profile. A random effects model was used for pooled data analysis. Heterogeneity and effect size were measured.

## 3. Results and Discussion

### 3.1. Effect of Structured Education

Structured education plays a key role in preventing hypoglycemia and other complications in people with diabetes who fast during the month of Ramadan. However, the majority of T2DM patients who fast do not consult their healthcare professionals before the commencement of Ramadan nor they monitor blood glucose level systematically throughout the month of Ramadan [20]. In regions with Muslims in minority, education of physicians on dose adjustment and patient risk stratification for fasting is uncertain. Also, physicians of such regions often lack sufficient knowledge about cultural aspects of fasting to provide adequate support to their patients. Eventually, patients seek advice of their relatives and religious scholars from the religious perspective [21]. In both scenarios, patients are at risk as neither physicians know the importance of religious fasting nor relatives or religious scholars know about potential medical complications of fasting. Physicians, religious scholars, and patients should work hand in hand for a safe Ramadan fast. One-on-one dietary educational session reduced the number of adverse events of fasting [22, 23]. Uysal et al. concluded that fasting is not contradictory to T2DM patients if appropriate advice about meals and use of glucose-lowering drugs are provided [24]. Most of the studies emphasized the importance of counselling before Ramadan fasting in terms of nutritional issues, timing and dosage of glucose-lowering medication, importance of self-monitoring of glucose, information on symptoms of hyperglycemia and hypoglycemia, and avoiding dehydration [11, 23, 25–30]. Studies also recommend collaboration of healthcare professionals for shared decision-making to resolve cultural perspective differences and unique cultural needs of patients [31]. A systematic review indicates empowering patients and healthcare professionals with the information of Ramadan fasting and disseminating the knowledge in major regional languages of the world to reach the information in underprivileged communities [32, 33]. Religious leaders and healthcare professionals play a pivotal role in providing information pertaining to safer fasting to the patients [7]. The onus of safe fasting lies with the people who intend to fast by visiting their physician for pre-Ramadan counselling and adhering to the recommendations of lifestyle advice on diet, medication regimen, cessation of smoking, and light physical exercise or sports [34]. One of the randomized controlled trials done on Ramadan-specific education coupled with telemonitoring compared with usual care found a decrease in the reported symptoms of hypoglycemia in the telemonitoring group, with only two participants out of forty-five reporting symptomatic hypoglycemia in comparison to eight participants out of eighty in the usual care group at the end of the Ramadan period (*p* = 0.04). Significant reduction in HbA1c was achieved in the telemonitoring group as compared to the usual care group at the end of the study (*p* < 0.01) [35]. McEwen et al. conducted a study to determine if individualized education before Ramadan resulted in a safe fast for people with T2DM as compared to usual care. The study indicated a lower incidence of reporting of severe hypoglycemia that required medical assistance, glucagon injection, or intravenous infusion of glucose in patients receiving individualized education as compared to patients receiving usual care (23% vs. 34%, *p* = 0.0017). The proportion of patients who were hospitalized after getting individualized education was much lower than the proportion of patients who received usual treatment (*p* = 0.0071). HbA1c improved by 0.7 ± 1.1% in the individualized education group versus 0.1 ± 1.3% (*p* < 0.0001) in the usual care group. The BMI values also corresponded to a decrease of 1.1 ± 2.4 kg/m^2^ vs. 0.2 ± 1.7 kg/m^2^ in the intervention and control groups (*p* < 0.0001) [23]. Further larger studies are required to increase the knowledge on dietary and treatment changes for proper advice to the T2DM patients who fast during Ramadan [36]. The review of literature on structured education thus indicated that T2DM patients can undergo safe fasting during the month of Ramadan if provided with appropriate pre-Ramadan advice on nutrition, physical activity, and management of glucose-lowering drugs.

### 3.2. Effects of Glucose-Lowering Drugs during Ramadan

Managing T2DM becomes a challenge during Ramadan especially as the dietary pattern and circadian rhythm changes drastically. Desired blood glucose level can be achieved through a proper attention to diet, physical activity, and possible modification of pharmacologic treatment. As Ramadan fasting may involve refraining oneself from food and water for even twelve hours or more from dawn to dusk, it becomes an essential need that guidance concerning the use and modification of treatment during this period should be individualized [37, 38].

Of the available glucose-lowering agents, the choice of treatment to an individual should be customized considering the pathogenesis of T2DM which is heterogenic in nature [39]. Glucose-lowering drugs available today can be classified into the following major classes: biguanides, sulphonylureas (SUs), dipeptidyl peptidase 4 (DPP-4) inhibitors, sodium-glucose cotransporter 2 (SGLT2) inhibitors, glucagon-like peptide-1 receptor (GLP-1) agonists, thiazolidinediones (TZDs), meglitinides, *α*-glucosidase inhibitors (AGIs), and insulin [40].

### 3.3. Biguanides

Metformin is widely preferred as the first-line glucose-lowering drug for the management of T2DM [40]. It is an insulin-sensitizing drug which exerts its effects by blocking liver gluconeogenesis thereby increasing the skeletal muscle uptake of glucose [41] but has also other potential modes of action. A pilot, open-label, observational study, conducted by Bonakdaran and Khajeh-Dalouie to determine the effects of fasting during Ramadan on glycemic excursions in patients with T2DM, reported that a remarkable difference was observed in the rate of hypoglycemic events (HEs) among the two groups of patients (patients administered metformin only versus patients administered SU). The percentage of HEs during Ramadan was found to be 0.11 ± 0.3% in the metformin group compared with 3.3 ± 3.8% in the SU group. The inclusion of SU in the treatment regimen increased the hypoglycemic risk of the study participants. The authors thereby concluded that in well-managed T2DM patients who are on metformin, the risk of hypoglycemia is minimal and patients may observe the Ramadan fast safely [27]. Generally, dosage modification is also not required with metformin. However, patients who take their usual dose at lunch may skip the dose during daytime fasting. A larger dose could be taken after breaking the fast while maintaining the morning dose as usual to prevent hyperglycemia. The dose can also be split into two: one-third can be taken at predawn while the rest at sunset [42, 43]. Though metformin once considered the first-line treatment for T2DM, many patients do not achieve glycemic control with this drug alone. A second drug is often needed to achieve glycemic control [41].

### 3.4. Sulphonylureas

SUs act by stimulating the insulin secretion by pancreatic *β*-cells and by decreasing the hepatic clearance of insulin. They are classified into two generations: first-generation SUs such as tolbutamide and chlorpropamide. Gliclazide, glipizide, glibenclamide, and glimepiride fall under the second generation of SUs [44]. As secretion of insulin is non-glucose-mediated, the traditional SUs result in a higher risk of hypoglycemia [45]. Zargar et al. evaluated the maintenance of glucose control with the intake of an evening dose of a long-acting SU in male T2DM patients fasting during Ramadan in Bangladesh, Pakistan, and India. These patients exhibited glycemic control with gliclazide-modified release (MR) 60 mg monotherapy and were switched to an evening dose only of the same drug during Ramadan and resumed their usual morning dose thereafter. The study results indicated an improvement in glycated hemoglobin (HbA1c) and blood lipids compared with levels prior fasting. None of the patients withdrew due to hypoglycemia. Side effects were observed in a few patients and medication compliance with once daily dosage was good. The authors thus concluded that the frequency of hypoglycemia and weight gain were negligible for male patients with gliclazide MR 60 mg monotherapy being switched to an evening dose schedule which enabled them to safely observe the Ramadan fast [46]. VIRTUE, a multicenter, prospective study, was conducted to assess the effect of vildagliptin relative to SUs in Muslim patients with T2DM observing the Ramadan fast. The study patients received treatment with vildagliptin or SU monotherapy or as an add-on to metformin. There was a significant and clinically relevant ~3.5-fold lower incidence of HEs with vildagliptin in comparison with SU treatment. Also, fewer patients experienced ≥1 HE with vildagliptin compared with SUs. Among the individual SU drug types, glipizide was the preferred drug of choice due to its lower incidence of HE (12.5%) followed by glimepiride (17.9%), gliclazide (19.2%), and glibenclamide (31.85%). A significant reduction in HbA1c was observed in the vildagliptin group as compared with the SU group. Minor reductions from prefasting baseline levels in body weight were noted in both cohorts. [47]. A similar study was conducted by Al Sifri et al. to evaluate the incidence of symptomatic hypoglycemia in Muslim T2DM patients fasting during Ramadan who were on SU before the study or switched to sitagliptin (with or without metformin). The percentage of patients recorded with either symptomatic or asymptomatic HEs was lower in the sitagliptin group (8.5%) as compared with the SU group (17.9%). Among the patients in the SU group, 6.6% under the gliclazide subgroup reported symptomatic HEs followed by glimepiride (12.4%) and glibenclamide (19.7%) [48]. Anwar et al. concluded that there is no statistically significant difference in the reported incidence of hypoglycemia between repaglinide and short acting SU glimepiride in T2DM patients during Ramadan [49]. The Glimepiride in Ramadan (GLIRA) study group concluded that the efficacy and safety of glimepiride in T2DM patients remained unchanged during the month-long daylight fast of Ramadan when the administration schedule of glimepiride was changed from morning to evening [18]. Cesur et al. conducted another multicenter study with glimepiride, repaglinide, and insulin glargine to compare their glycemic effects in T2DM patients during Ramadan fasting. Patients were divided into fasting and nonfasting groups, and metformin was administered to all patients. Hypoglycemia was reported in 14.3% of patients in the glimepiride group followed by 11.1% in the repaglinide group and 10% in the insulin glargine group, but these differences among the drug groups were not significant. The levels of HbA1c did not show any remarkable change in either the fasting or non-fasting group. The fructosamine levels presented a notable increase at 1-month post-Ramadan compared with pre-Ramadan and post-Ramadan in both the fasting and nonfasting groups with no significant difference between the three drug groups. No changes were reported in the fasting group in body mass index (BMI) and plasma lipids [50]. Another multiregional, double-blind study randomized 557 patients with T2DM who were previously on metformin and any SU to receive either vildagliptin or gliclazide plus metformin. The percentage of hypoglycemia was not different between the vildagliptin group (6.0%) and the gliclazide group (8.7%) [51]. Newer generation SUs, especially gliclazide, seem to be safer in comparison to the older generation of SUs, owing mainly to their lower risk of HEs [44].

### 3.5. Dipeptidyl Peptidase 4 Inhibitors

Hypoglycemia and hyperglycemia remain the two major concerns associated with Ramadan fasting, and therefore, the treatment options should be aimed at reducing these risks in patients who intend to fast [52]. The DPP-4 inhibitors have a glucose-dependent mechanism of action and inhibit the disintegration of GLP-1 leading to an increase in its systemic concentration and secretion of endogenous insulin. This process in result reduces the secretion of glucagon [53]. Sitagliptin, the first drug from this class, was approved more than a decade ago by the United States Government's Food and Drug administration. This was followed by several other DPP-4 inhibitors such as vildagliptin, saxagliptin, linagliptin, and alogliptin which are currently available. Vildagliptin and sitagliptin are the most frequently studied DPP-4 inhibitors to assess the safety and efficacy in T2DM patients during Ramadan [54, 55]. The use of DPP-4 inhibitors has increased in the recent times due to its inherent potential to decrease the levels of blood glucose, along with an added advantage of good tolerability and reduced hypoglycemic risk [9, 53]. A study conducted by Hassoun et al. assessed the impact of vildagliptin in relation to SU on hypoglycemic occasions and concluded that the vildagliptin cohort reported a larger reduction in HbA1c and body weight from baseline to the end of the study as compared with the SU cohort during the Ramadan fasting period [56]. A 4-week, open-label, observational study comparing vildagliptin and SU with or without metformin did not exhibit any significant differences in HEs among the two groups. However, significant reductions in HbA1c level and body weight were observed in the vildagliptin cohort. Neither group exhibited drug-related serious adverse event or discontinuation of treatment due to adverse events [57]. Malha et al. conducted a randomized open-label clinical trial to determine the glycemic effects of vildagliptin in patients with T2DM before, during, and after Ramadan fasting. The SU group showed a numerically higher incidence of hypoglycemia during Ramadan compared with the vildagliptin group (26 versus 19, *p* = 0.334). The BMI value was higher at baseline in the vildagliptin group compared to post-Ramadan (29.5 Kg/m^2^ versus 28.9 Kg/m^2^) whereas contrasting values were noted in the SU group (28.9 Kg/m^2^ baseline versus 29.8 Kg/m^2^ post- Ramadan) [58]. A study by Halimi et al. assessed the rate of hypoglycemia during Ramadan in patients with T2DM treated with their usual dual therapy of metformin-vildagliptin or metformin SU/insulin secretagogue (IS). A minimum of single episode of symptomatic hypoglycemia was seen in 37.2% of patients in the IS cohort versus 34.2% in the vildagliptin cohort. Severe hypoglycemia was also more evident in the IS group (10.4%) as compared to the vildagliptin group (2.6%). Glycemic and weight control were not different in these cohorts [59]. Based on the few studies available thus far, DPP-4 inhibitors appear to be effective in improving glycemic control with lower rates of hypoglycemia during fasting, making them a convenient treatment option during Ramadan.

### 3.6. Sodium-Glucose Cotransporter 2 Inhibitors (SGLT2is)

SGLT2is act by inhibiting the absorption of glucose from the proximal convoluted tubule of the kidney thereby promoting the excretion of glucose in urine [60]. Commonly available drugs under this class include dapagliflozin, canagliflozin, ipragliflozin, empagliflozin, and ertugliflozin [54, 61]. Two studies assessed the safety and efficacy of SGLT2i and reported very few patients experiencing hypoglycemia in the SGLT2i group as compared with those treated with SUs. Though treatment with SGLT2i was generally well tolerated, patients were more prone to develop volume depletion symptoms such as dehydration in this group when compared to the SU group [62, 63]. More studies are therefore required to assess the safety and efficacy of SGLT2i especially for their use in Ramadan.

### 3.7. Glucagon-Like Peptide-1 (GLP-1 Agonists)

GLP-1 agonists that are incretin mimetics are increasingly used in the treatment of T2DM in recent years. They act by binding to the GLP-1 receptor, thereby reducing the glucagon concentration with improved insulin sensitivity. They also act by delaying gastric emptying, increasing satiety, and decreasing free fatty acid concentrations and body weight. Several drugs in this class have been developed [64]. The most commonly studied GLP-1 agonists for their safety and efficacy in T2DM patients during the month of Ramadan are exenatide and liraglutide. A study compared liraglutide and SU, both in combination with metformin, during Ramadan and reported 2% of patients in the liraglutide group experienced hypoglycemic episodes as compared to 11% of patients in the SU group. The trial also reported that body weight reduced more with the liraglutide group (*p* = 0.0091). The fructosamine levels were similar in both groups from beginning to the end of Ramadan (liraglutide −12.8 *μ*mol/L vs. SU −16.4 *μ*mol/L). However, there was a significant reduction in fructosamine levels with liraglutide versus SU from baseline to the beginning of Ramadan (*p* = 0.0024) [65].

A triple-blind, placebo-controlled study was performed by Buse et al. to evaluate the glycemic control of exenatide in T2DM patients treated with SU. Subjects were randomized to 5 *μ*g or 10 *μ*g subcutaneous exenatide or placebo twice daily in two different groups. The subjects followed their usual SU therapy for a period of at least three months before screening. The study concluded that long-term use of exenatide at fixed subcutaneous doses of 5 *μ*g and 10 *μ*g twice daily appears to be an effective treatment option for patients with T2DM who are not adequately managed with SUs [66]. Literature to evaluate the safety and efficacy of exenatide in T2DM patients who fast during Ramadan is scanty, and hence, more studies are welcome in this area.

### 3.8. Thiazolidinediones

TZDs act by improving the insulin sensitivity and increasing the insulin stimulated glucose with negligible effect on the hepatic glucose output. The signaling of insulin is stimulated in vitro with the assistance of muscle culture cells. Moreover, they are known to diminish circulating free fatty acid levels leading to insulin resistance [67]. Drugs under this class include troglitazone, rosiglitazone, and pioglitazone. Studies evaluating the effects of TZDs in T2DM patients are limited. A double-blind, randomized controlled trial was conducted by Vasan to evaluate the effects of pioglitazone in Muslim patients fasting during Ramadan reported remarkable improvement in glycemic control when pioglitazone was used as an add-on therapy with other oral glucose-lowering drugs (SUs, metformin, meglitinides and acarbose) without any increase in HE. However, significant weight gain was observed with the pioglitazone group. Unlike other classes of drugs, dose adjustment is not required with pioglitazone [68]. Although this class of drugs are safe to use in T2DM patients during Ramadan due to their lower risk of hypoglycemia, not many studies are available to confirm the same. The availability of pioglitazone is also restricted in few countries, which eventually has led to fewer studies being conducted.

### 3.9. Meglitinides

This class of drugs belongs to insulin secretagogues which increase the secretion of insulin similar to the action of SUs but has a shorter half-life [69]. Repaglinide and nateglinide are the commonly studied drugs under this class. A study to compare the treatment efficacy of repaglinide versus glimepiride was conducted in T2DM patients fasting during Ramadan, by Anwar et al. The study inferred that glycemic control was better controlled in the glimepiride group as compared to the repaglinide group. However, there was no statistically significant difference with respect to the incidence of hypoglycemia between the two groups. In comparison to repaglinide, the study concluded that glimepiride may be a better choice of drug during Ramadan due to its longer duration of action [49]. Another study was carried out by Bakiner et al. to evaluate the efficacy of repaglinide three times a day along with a single dose of insulin glargine. The participants were grouped under the fasting and nonfasting groups. The results showed that glycemic control did not change during this period. Also, none of the participants from the fasting group reported a HE. Thus, combining repaglinide with insulin glargine was proved to be safer in T2DM patients who fast in the month of Ramadan [70]. More studies are required to confirm the safety of this class of drugs.

### 3.10. *α*-Glucosidase Inhibitors

AGIs act by inhibiting the enzymes that are responsible for the conversion of complex nonabsorbable carbohydrates into simple absorbable carbohydrates. This process delays the digestion rate, thereby leading to a reduction in the postprandial glucose (PPG) and levels of insulin. Acarbose, miglitol, and voglibose are the drugs that fall under this class [71]. The review of the existing literature indicates a lower risk of hypoglycemia with this class of drugs and can be used without any dosage adjustment. However, gastrointestinal side effects remain the cause of concern [8, 9, 42, 43, 53]. Currently, there are no randomized control trials available that have investigated the use of AGIs in T2DM patients fasting during the Ramadan period. They can be used in Ramadan as monotherapy mainly due to their little chances of causing hypoglycemia; however, the treatment should be individualized.

### 3.11. Insulin and Its Analogs

Insulin is generally indicated in patients who are intolerant to one or more oral glucose-lowering drugs, who do not exhibit adequate glycemic control with oral monotherapy, and who personally prefer insulin over other oral glucose-lowering agents. An open-label randomized clinical trial by Hassanein et al. to compare the safety and efficacy of insulin degludec/insulin aspart (IDegAsp) and biphasic insulin aspart 30 (BIAsp 30) was conducted in T2DM patients fasting before, during, and after Ramadan. The treatment initiation period was fixed to a minimum of 8 weeks before the commencement of Ramadan. The dose of insulin was decreased at suhoor by 30–50% on the first day of Ramadan and readjusted back to pre-Ramadan levels at the end of the month. The patients were observed for another 4 weeks (post-Ramadan period). The treatment period witnessed similar glycemic efficacy in both arms. There was no significant difference observed between the two groups in terms of HbA1c reduction and change of fructosamine levels from baseline to end of Ramadan or end of 4 weeks post-Ramadan. Throughout the treatment period, the overall rate of hypoglycemia was significantly lower in the IDegAsp arm in comparison with the BIAsp 30 arm (*p* < .0001) [72]. The Ramadan study group compared Insulin lispro Mix25™ with human insulin 30/70 to determine their impact on morning and evening PPG control and on average daily blood glucose in T2DM patients fasting during Ramadan. All patients were administered human insulin 30/70 during the lead-in period. At visit 2, the patients were administered with Insulin lispro Mix25 (Humalog R Mix25™) for the initial two weeks of Ramadan, followed by therapy with human insulin 30/70 for the remaining two weeks or vice versa. Though the numbers of hypoglycemic episodes were reported to be similar in both the groups, the treatment with lispro Mix25 was associated with a lower average daily glycemia as compared with human insulin 30/70. There was no significant change in body weight reported in either group [73].

### 3.12. Meta-Analysis

The main objective of this meta-analysis was to compare the safety profile of DPP-4 inhibitors versus SU in T2DM patients fasting during Ramadan. The primary outcome analyzed for this purpose was the number of confirmed symptomatic HEs that occurred during the fasting period. The statistical analysis was carried out using Review Manager version 5.3. Odds ratio was calculated to compare the degree of fasting risk in T2DM patients with 95% Confidence Interval (CI) using the Mantel-Haenszel method. A random effects model was applied to determine the effect size.

Seven papers, with a total of 1723 participants, were included in this meta-analysis [47, 48, 51, 57, 58, 74, 75]. The sample size ranged between 30 and 669. Patients with T2DM age 18 or older were included. One study used sitagliptin [48] while the other six used vildagliptin [47, 51, 57, 58, 74, 75]. The seven studies compared DPP-4 with SU (glimepiride, gliclazide, glibenclamide, or glipizide). All the seven studies assessed HEs during the fasting period. Six studies assessed changes in HbA1c and body weight apart from HE [47, 51, 57, 58, 74, 75].

**Figure 2:**
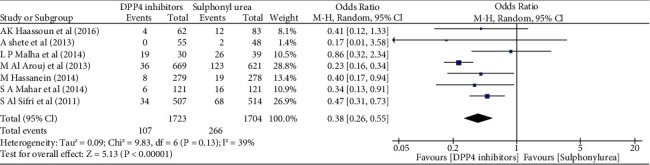
Forest plot for hypoglycemic event comparing DPP-4 inhibitors and sulphonylurea.

The number of hypoglycemic episodes with DPP-4 inhibitors was considerably lower compared to with SUs (107 vs. 266) (Figure 2). The meta-analysis indicates DPP-4 inhibitors would significantly reduce the risk of hypoglycemia as compared to sulphonylurea (odds ratio = 0.38, 95% CI: 0.26 to 0.55, *p*˂0.00001). Among the DPP-4 inhibitors, vildagliptin was found to be an effective, safe, and well-tolerated regimen with a low incidence of hypoglycemia accompanied by good glycemic control. Physicians also recommend the use of vildagliptin over SU for use in high-risk populations such as the elderly. One of the studies reported that the use of a sitagliptin-based regimen decreased the risk of hypoglycemia compared with a SU-based regimen. Among the SUs, gliclazide was found to be much safer with a lower incidence of hypoglycemia as compared to other listed drugs in this category [48]. This meta-analysis concluded that DPP-4 inhibitors remain the preferred drug class among T2DM patients fasting when compared with SUs. One previous meta-analysis conducted by Gray et al. also reached at the same conclusion [76].

## 4. Limitations

The main limitation is that meta-analysis included all types of study design. Differences in the reporting methods of hypoglycemia whether it is hypoglycemia incidence, hypoglycemia event, or symptoms in the studies are included. This can lead to publication bias as symptoms are subjective to an individual. Further, our study evaluated only patients with T2DM who wish to fast during Ramadan, and thus, the conclusion cannot be generalized to T1DM patients. We performed the extraction of literature for review from only one search engine (PubMed). Publication bias could not be established as the number of articles included for meta-analysis is less than 10. However, visual inspection of the funnel plot indicates a slight asymmetry which might be subjective.

## 5. Conclusions

Our review indicates that structured education and counselling play an important role in preventing the complications associated with Ramadan fasting in T2DM patients. Structured education and diabetes treatment program during Ramadan in patients with T2DM who wish to fast, empowered patients to self-manage diabetes, lose weight, improve glycemic control, and avoid other complications of fasting. Ramadan-specific structured education along with telemonitoring also decreased the symptomatic hypoglycemia in T2DM patients, thus achieving safer fast during Ramadan. Early and effective counselling at least one to two months prior to Ramadan with a particular emphasis on blood glucose measurement, information about signs and symptoms of hyperglycemia and hypoglycemia, changes in meal pattern, physical activity, and medications is recommended according to the risk stratification of the T2DM patients willing to fast to avoid complications during Ramadan. A patient-focused educational program that encourages optimal health care during the fasting period is the need of the hour. Both the healthcare providers and T2DM patients are recommended to have customized education preferably in their regional language for a safe Ramadan fasting. Regarding the choice of glucose-lowering drugs during Ramadan, DPP-4 inhibitors are the preferred class because of their safety and efficacy. Our meta-analysis corroborates this finding. However, we recommend assessing the safety and efficacy profile of other classes of drugs such as SGLT2is, TZDs, meglitinides, and AGIs through randomized clinical trials as the literature on the use of drugs in these categories during Ramadan is scarce.

## Data Availability

The data supporting this systematic review and meta-analysis are from previously reported studies and datasets, which have been cited.

## Abbreviations

AGIs: *α*-Glucosidase inhibitors
BMI: Body mass index
DPP-4: Dipeptidyl peptidase 4
GDP: Gross Domestic Product
GLP-1: Glucagon-like peptide-1
HbA1c: Glycated hemoglobin
HE: Hypoglycemic event
IDF-DAR: International Diabetes Federation-Diabetes and Ramadan (DAR) International Alliance
MENA: Middle East and North Africa
MESH: Medical Subject Headings
PICOS: Population Intervention Comparison Outcome Study Design
PPG: Postprandial glucose
PRISMA: Preferred reporting items for systematic reviews and meta-analysis
SU: Sulphonylurea
SGLT2i: Sodium-glucose cotransporter 2 inhibitor
T1DM: Type 1 Diabetes Mellitus
T2DM: Type 2 Diabetes Mellitus
TZDs: Thiazolidinediones.

## Supplementary Materials

Supplementary 1 Table 1: characteristics of included studies.

Supplementary 2 Table 2: characteristics of classes of drugs and studies.
