# Supplementary material for: Safety Assessment of Glucose-Lowering Drugs and Importance of Structured Education during Ramadan: A Systematic Review and Meta-Analysis
Source: J Diabetes Res. 2022 Feb 18;2022:3846253. doi: 10.1155/2022/3846253 (PMC8886741; doi:10.1155/2022/3846253)
Supplement: Supplementary 1 — Table 1: characteristics of included studies. [file 3846253.f1.docx]

**Table No: 1 Characteristics of included studies**

| **Sr. No** | **Title** | **Type of Diabetes** | **Type of Study/Country** | **Drug involved** | **Conclusion** |
| --- | --- | --- | --- | --- | --- |
| 01 | Fasting in Ramadan of Muslim patients with diabetes Mellitus, and knowledge and practice in relation to diabetes control in Brunei | DM | Epidemiology study,  Brunei | None | Diabetes education and early treatment adjustment especially before and during the month of Ramadan is essential to minimize hypoglycaemic risks and frequency of diabetes complications. |
| 02 | Ramadan fasting with diabetes: an interview study of inpatients' and general practitioners' attitudes in the South of France | T1DM & T2DM | Cross-sectional population based study,  France | OAD exclusively, OAD+insulin, insulin only and insulin total | Systematic advice on treatment adjustment is required. Because of the religious nature of the fast, the decision is ultimately up to the patient, but a holistic approach should reduce the gap in the physician–patient relationship. New therapeutic strategies, including glucagon-like peptide-1 (GLP-1) analogues, dipeptidyl peptidase (DPP)-4 inhibitors for type 2 diabetics and multiple injection basal–bolus regimens for type 1 diabetics, should reduce the number of adverse events during the fast and facilitate therapeutic adjustments. Bilingual translators and Imams should also be included. Medical training for GPs and Imams on the subject of Ramadan fasting. |
| 03 | Dietary patterns and glycemic control and compliance to dietary advice among fasting patients with diabetes during Ramadan | DM | Observational study, Pakistan | - | The diet of patients with diabetes should  be assessed prior to Ramadan. Structured Ramadan-specific dietary advice should be given to every individual with diabetes who intends to observe the fast. |
| 04 | Clinical and metabolic effects of fasting in 41 type 2 diabetic patients during Ramadan | T2DM | Observational study, Turkey | Some patients were on a single oral hypoglycemic agent (OHA) and some were on combined OHAs (OHA not specified) | No statistically significant change was observed in mean body weight, BMI, total cholesterol level, or LDL cholesterol level. The mean HDL cholesterol level increased significantly during Ramadan, and it was still significantly higher 3 weeks after Ramadan than that before Ramadan. The mean triglyceride level was significantly lower during the 4^th^ week of Ramadan than it was before Ramadan. Patients, on a fixed caloric intake, did not gain or lose weight. Nobody had reported severe hypoglycemia or neuroglycopenia in patients on OHA therapy  during Ramadan. In the whole group, a slight deterioration in HbA1 c that returned to the initial values early after Ramadan. Eight of our  patients had increased weekly incidence of  symptoms attributable to low blood glucose  that disappeared after Ramadan. The study concluded that type 2 diabetes is not a contraindication to fasting in Ramadan. Type 2 diabetic patients on single or combination OHAs could observe Ramadan fasting with appropriate instruction about meals and OHA use. However, some patients may still experience an increased number of hypoglycemic episodes. |
| 05 | Knowledge of diabetes mellitus and safe practices during Ramadan fasting among Muslim patients with diabetes mellitus in Singapore | DM | Survey/Questionnaire, Singapore | DM treatment consisted of insulin therapy, oral glucose-lowering drugs, and combined therapy | There is a need for a focused educational and interventional programme that encourages optimal patient care during this critical period. For example, a programme that educates both Muslim patients with DM and healthcare providers on how to manage complications that could occur during fasting should be developed. The development of public health programmes that specifically address DM management when fasting are especially important in countries where a significant proportion of diabetic patients practice fasting during Ramadan. |
| 06 | Exploring Drug-Related Problems in Diabetic Patients during Ramadan Fasting in Saudi Arabia: A Mixed-Methods Study | DM | Mixed methods approach (Qualitative and Quantitative), Saudi Arabia | - | The study has identified two potential risk factors for DRPs: patient-related factors  including changes in medicines intake during fasting and healthcare professionals-related factors including lack of advice from healthcare professionals regarding fasting. Over just half or the participants reported to have received advice about fasting from healthcare professionals that highlights the lack of effective communication between healthcare professionals and patients. Patients reported that pharmacists’ role was limited to supply of medicines only and did not extend to the provision of advice related to the use of medicines or the management of condition. The self-alteration of medicines schedule by patients without receiving advice from healthcare professionals can put the patients at risk of developing potential DRPs. The healthcare professionals including pharmacists should therefore need to actively engage with the patients who are at risk of DRPs by providing Ramadan specific education and counseling. This study has identified several potential risk factors for DRPs that can help healthcare professionals in developing comprehensive educational programs for patients with long-term medical conditions such as diabetes. Patients should be advised to seek detailed advice from their healthcare professionals about the benefits and risks associated with fasting. Patients who insist on fasting should be provided explicit education about the schedule of their medicine intake during fasting, self-monitoring of blood glucose levels, recognition of signs and symptoms of low or high blood glucose levels and importance of undertaking regular physical activity to minimize the incidence of general health or DRPs. |
| 07 | The effects of fasting during Ramadan on glycemic excursions detected by continuous glucose monitoring system (CGMS) in patients with type 2 diabetes | T2DM | Pilot, observational study, Iran | Sulfonyl urea, Metformin | The study recruited well controlled diabetic patients. Despite good control of diabetes in these patients, CGMS results showed frequent hyperglycemic and hypoglycemic episodes in them one month before Ramadan. There was a significant reduction of hyperglycemic events during Ramadan. An improvement of glycemic control was also noted during Ramadan. The extent and duration of hypoglycemic events was not significantly different between two states (Ramadan and before). A significant increase in number of hypoglycemic events in Ramadan was found in patients who take sulfonylurea compared with those who take only metformin. It seems that the addition of sulfonylurea, increases the risk of hypoglycemia and patients should be strongly advised for risk of hypoglycemia during Ramadan. Patients have to receive an appropriate education including frequent blood glucose measurement, information about sign and symptoms of hyperglycemia and hypoglycemia, changes in meal pattern, physical activity and medications. |
| 08 | Fasting during Ramadan and Associated Changes in Glycaemia, Caloric Intake and Body Composition with Gender Differences in Singapore | T2DM | Prospective cohort study, Singapore | Oral agents, insulin therapy | The study shows that daylight fasting during the month of Ramadan appears to confer benefits on glycaemia and body composition, especially among women with type 2 diabetes, with improvement in HbA1c and a reduction in VFA. In addition, fasting during Ramadan is safe in a group of individuals who have received prior education on dietary counselling, glucose monitoring, and adjustment of medication dosage and timing. None of the subjects required hospitalization for any of the acute complications of diabetes, such as hyperglycemic emergencies (diabetic ketoacidosis or hyperosmolar hyperglycemic state), hypoglycemia or dehydration. Symptomatic hypoglycemia occurred in few subjects, and these were self-managed by the subjects with subsequent adjustment of medication dose by the physician. Healthcare professionals should also actively ask about the intention to fast among their Muslim patients with diabetes. Nutritional and dietary counselling is an essential component in the education package. Healthier alternatives to sugary fluids and sweet foods traditionally used to break fast should be advised to Muslims with diabetes along with avoidance of high saturated fat meals. Ramadan fasting confers metabolic and glycemic benefits (albeit modest) and can be practiced safely with appropriate patient education, blood glucose monitoring, adjustment of medication dose, and timing among Muslims with diabetes. |
| 09 | Muslims with non-insulin dependent diabetes fasting during Ramadan: treatment with glibenclamide | T2DM | Non-randomised control group of patients who did not fast during Ramadan and two groups of  patients who fasted randomised equally to one of two  regimens: to take their usual morning dose of glibenclamide  in the evening and their usual evening dose  before dawn, or to follow this pattern but to reduce the total dose by a quarter.  Morocco | Glibenclamide | Glibenclamide treatment was effective  and safe for patients with non-insulin dependent diabetes who fasted during Ramadan compared with non-fasting patients in the same environment. In particular, no excess of hypoglycaemic events were  reported during fasting compared with before and after Ramadan. The increases in creatinine concentrations although  still within normal limits, suggest that doctors should advise their fasting patients to drink more fluid during Ramadan. No measurable impact on glycaemic control reported. It was concluded to switch the morning dose (together with any midday dose) of glibenclamide with that taken at sunset. |
| 10 | The puzzle of self-reported weight gain in a month of fasting (Ramadan) among a cohort of Saudi families in Jeddah, Western Saudi Arabia | T2DM | A Cross-section study using a pre-designed questionnaire, Saudi Arabia | - | It is found in this study that weight gain and not weight loss was likely to happen after Ramadan by Saudis living in Jeddah. The findings in this small-scale cross-sectional  study indicates the need for large scale studies at national levels not only in Saudi Arabia but also in adjacent gulf countries and perhaps MENA countries of similar cultural and social back grounds. There is need  for educational programs which focus on healthier life-style in Ramadan and dietary modification of a population which reports one of the highest prevalence rates of  diabetes. |
| 11 | Patients with diabetes observing Ramadan: The experience of Muslims in the United States | T2DM | Exploratory design, semi-structured interviews, United States of America | - | Healthcare providers need to hold conversations embracing shared  decision-making to resolve healthcare dilemmas resulting from cultural differences, expand cultural knowledge, and adapt services to meet culturally-unique needs of their patients. |
| 12 | Telemonitoring in fasting individuals with Type 2 Diabetes Mellitus during Ramadan: A prospective, randomised controlled study | T2DM | Randomized control study, Malaysia |  | The study used telemonitoring intervention in diabetes management during Ramadan in a community setting. Observed that it was practical to implement Ramadan-specific education coupled with telemonitoring  as a supplement to current diabetes care management for individuals who fast during this period. Individuals with type 2 diabetes mellitus who intend to fast should undergo a medical assessment at least 1 month  prior to the month of Ramadan and receive an education that is tailored towards Ramadan fasting. A remote alert that reports low hypoglycemic levels should be implemented to allow appropriate intervention should a hypoglycemic event occurs. At the end of the Ramadan period, reported symptomatic hypoglycemia was lower in participants in the telemonitoring group compared to the usual care group. There was a significant decrease in mean HbA1c in the telemonitoring group compared to a decrease in the usual care group at the end of the study. More participants in the telemonitoring group achieved HbA1c level of ≤ 7.0% compared to the usual care group. The telemonitoring group also reported significant improvements in lipid control at the end of Ramadan. An improvement in the quality of life was also noted, albeit not achieving statistical significance. No significant changes in other secondary outcome measures including blood pressure, weight, diabetes distress assessment, and diabetes self-efficacy scale were noted. |
| 13 | Ramadan fasting: a study of changes in glucose profiles among patients with diabetes using continuous glucose monitoring | T2DM | Prospective observational study, United Arab Emirates | Medication ranged from none to a  mixture of oral agents including a sulphonylurea,  including sitaglitin or vildagliptin, with or without insulin, exenatide alone and  exenatide plus oral agents. | During Ramadan, changes to medication followed American Diabetes Association guidelines and included a 20–30% reduction in dose in patients on sulphonyl ureas or insulin. There was no episode of symptomatic hypoglycemia during Ramadan fasting, although CGM recorded at least one hypoglycemic event (≤70 mg/dL) in 14  patients. The study further underlines the importance of focused pre-Ramadan counseling for patients with diabetes specifically addressing nutritional issues as well as timing and dose changes to antidiabetic medication. Further and larger studies of Ramadan fasting can improve the knowledge base for such advice on dietary and treatment changes. |
| 14 | Sulphonylureas in the management of type 2 diabetes during the fasting month of Ramadan | T2DM | Questionnaire (Asia: India, Bangladesh & Pakistan) | Sulphonyl urea (Gliclazide) | Male patients under glycaemic control with gliclazide MR 60 mg monotherapy can safely maintain the same degree of control by switching to an evening dose schedule during Ramadan. The frequency of hypoglycaemia is low, and weight gain minimal. |
| 15 | The effect of vildagliptin relative to sulphonylureas in Muslim patients with type 2 diabetes fasting during Ramadan: The VIRTUE study | T2DM | Multicentre, post-authorisation, prospective  study conducted in countries in the (Middle East and Asia: Bangladesh, Egypt, India, Indonesia, Kuwait, Lebanon, Oman, Pakistan, Saudi Arabia and the United  Arab Emirates) | Villdagliptin  or SU as add-on to metformin or as monotherapy | Vildagliptin therapy was associated with significantly fewer patients experiencing hypoglycaemia compared with SU therapy in this large representative cohort of fasting Muslim patients with T2DM. This outcome is particularly meaningful when viewed in the context of good glycaemic and weight control observed in vildagliptin-treated patients who fasted in this study. Vildagliptin was well-tolerated in this patient population. |
| 16 | The incidence of hypoglycaemia in Muslim patients with type 2 diabetes treated with sitagliptin or a sulphonylurea during Ramadan: a randomised trial | T2DM | Open-label study (Asia, Africa & Middle East: Egypt, Israel, Jordan, Lebanon, Saudi Arabia and the United Arab  Emirates) | Sulphonylurea [glimepiride, gliclazide (immediate  or modified release) or glibenclamide (glyburide)]  with or without metformin | Switching treatment to a sitagliptin- based regimen decreased the risk of hypoglycaemia compared with remaining on a sulphonylurea-based regimen. In this study, the incidence of hypoglycaemia was lower with gliclazide relative to the other sulphonylurea agents and similar to that observed with sitagliptin. Both treatment regimens were generally well tolerated during the month of Ramadan. |
| 17 | An open label comparative study of glimepiride versus repaglinide in type 2 diabetes mellitus Muslim subjects during the month of Ramadan | T2DM | Open label Comparative Study, Malaysia | Short acting SU (repaglinide) or a long  acting SU (glimepiride). | Muslims with type 2 diabetes mellitus  treated with glimepiride showed a trend towards better glycaemic control than patients treated with repaglinide during Ramadan. There have been no reports of severe hypoglycemia in patients on OHA therapy during Ramadan. Nevertheless, the decision to prescribe anti-diabetic medication during Ramadan should be individualised. |
| 18 | The efficacy and safety of glimepiride in the management of type 2 diabetes in Muslim patients during Ramadan | T2DM | Open-label, prospective,  observational study, (Africa, Asia & Middle East: Algeria, Egypt, Indonesia, Jordan, Lebanon, and Malaysia) | Glimepiride | The results show that the efficacy and safety of glimepiride in type 2 diabetic patients is not altered during the month-long daylight fast of Ramadan, when the time of administration of glimepiride is changed from the morning to the evening. Muslim type 2 diabetic patients taking glimepiride who are normally well controlled can fast during Ramadan if they wish to do so, with no  deterioration of glycemic control. |
| 19 | A comparison of glycemic effects of glimepiride, repaglinide,  and insulin glargine in type 2 diabetes mellitus  during Ramadan fasting | T2DM | Open-label, multicenter, prospective, observational  Study, Turkey | Metformin, glimepiride, repaglinide and insulin glargine | 14.3% of patients in the glimepiride group reported hypoglycemia followed by 11.1% in the repaglinide group and 10% in the insulin glargine group. However, no significant difference was observed between the fasting groups. The levels of HbA1c did not exhibit any remarkable change in either of the fasting or non-fasting group. The fructosamine levels presented a notable increase at 1-month post-Ramadan compared to pre-Ramadan and post-Ramadan in both fasting and non-fasting groups. However, between the three drug groups there was no significant difference noted. No changes were reported in the fasting group with respect to BMI and plasma lipids. |
| 20 | A double-blind, randomized trial, including frequent patient-physician contacts and Ramadan-focused advice, assessing vildagliptin and gliclazide in patients with type 2 diabetes fasting during Ramadan: the STEADFAST study | T2DM | Randomized Controlled double blinded trial  (2014): (Middle East, Europe, and Asia 69 sites in 16 countries) | Vildagliptin and gliclazide plus metformin dual therapy | Vildagliptin in combination with metformin is an effective and well-tolerated treatment option in patients with T2DM fasting during Ramadan, with a low incidence of hypoglycemia and similar efficacy to the SU gliclazide in combination with metformin. Vildagliptin was also associated with good treatment adherence, as well as a small decrease in body weight and the study did not identify any safety signals or unforeseen risks in vildagliptin-treated patients with T2DM fasting during Ramadan. Gliclazide in combination with metformin also showed a low incidence of hypoglycemia. Although numerically still higher than with vildagliptin, in particular when assessing confirmed events, the difference between the two treatments did not reach statistical significance. The positive effect of gliclazide is suggested to be linked to the specific circumstances of this study, including frequent patient–physician contacts, Ramadan-focused advice, a recent switch in treatment, and very well-controlled patients, which is different from what is often seen in real life. |
| 21 | The effect of vildagliptin relative to sulfonylurea as dual therapy with metformin (or as monotherapy) in Muslim patients with type 2 diabetes fasting during Ramadan in the Middle East: the VIRTUE study | T2DM | Multicenter, prospective observational cohort study (Middle East: United Arab Emirates, Kuwait, Lebanon, Oman and Saudi Arabic) | Vildagliptin, sulfonyl urea as dual therapy with metformin | A significant and clinically relevant seven-fold lower incidence of hypoglycemic events with vildagliptin versus SU treatment was reported in this large cohort of fasting patients with T2DM from the Middle East Region. Good glycemic and weight control was observed in the vildagliptin treated patients. This was accompanied by favorable tolerability and safety observed in vildagliptin treated patients. Vildagliptin might be a useful choice of therapy for patients with type 2 diabetes mellitus, particularly high-risk populations such as the elderly, fasting during Ramadan. |
| 22 | Vildagliptin vs sulfonylurea in Indian Muslim diabetes patients fasting during Ramadan | T2DM | Non-interventional, open-label, observational study, (India) | Glibenclamide, Gliclazide, Glimepiride &  Glipizide | No significant difference reported in hypoglycemic events in the two groups (Vildagliptin vs Sulfonyl urea) with or without metformin. Significant reduction in HbA1c levels and body weight were observed in the vildagliptin cohort. Neither of the group exhibited drug related SAEs or discontinuation due to AE. |
| 23 | Glycemic effects of vildagliptin in patients  with type 2 diabetes before, during and after the period of fasting in Ramadan | T2DM | Randomized open-label clinical trial (Lebanon) | Metformin,  Glimepiride/Gliclazide & Vildagliptin | The sulfonyl urea group showed a higher incidence of hypoglycemia during Ramadan as compared to the Vildagliptin study group (26 versus 19) however, this was not statistically significant *p* = 0.334. A similar change was also reported for HbA1c values in both the groups. The BMI value was higher at baseline in the vildagliptin group compared to post Ramadan (29.5 versus 28.9) whereas contrasting values were noted in the sulphonyl urea group (28.90 baseline versus 29.8 post Ramadan) |
| 24 | Experience with Vildagliptin in Type 2 Diabetic Patients Fasting During Ramadan in France: Insights from the VERDI Study | T2DM | Prospective, non-interventional study, France | Metformin &  Vildagliptin | A minimum of single episode of symptomatic hypoglycemia was seen in 37.2% of patients in the IS cohort (metformin sulfonylurea/glinide) versus 34.2% in the vildagliptin cohort. Severe hypoglycemia was also more evident in the IS group (10.4%) as compared to the vildagliptin group (2.6%). Glycemic and weight control was reported to be alike in both the cohorts. |
| 25 | Switching from sulphonylurea to a sodium-glucose cotransporter2 inhibitor in the fasting month of Ramadan is associated with a reduction in hypoglycaemia | T2DM | Randomized open label  Two arm parallel group, Malaysia | Sulphonylurea (glimepiride,  gliclazide or glibenclamide) and metformin  Intervention group: 10mg of  dapagliflozin daily  Control group: Sulphonylurea. Both group receiving background metformin  therapy | In combination with metformin,  dapagliflozin was associated with a lower risk of hypoglycaemia than sulphonylurea. Greater reduction in reported symptomatic hypoglycaemia, from 24.1 to 3.4%,  was observed when patients were switched from sulphonylurea to dapagliflozin. No difference in overall adverse events was observed between the dapagliflozin and sulphonylurea groups. The study suggest that switching from sulphonylurea to dapagliflozin is associated with a lower risk of hypoglycaemia while retaining a similar glycaemic efficacy. Thus, this can be considered an alternative approach for preventing hypoglycaemia during the fasting month of Ramadan. |
| 26 | Tolerability of canagliflozin in patients with type 2 diabetes mellitus fasting during Ramadan: Results of the Canagliflozin in  Ramadan Tolerance Observational Study (CRATOS) | T2DM | Non-randomised,  parallel-cohort,  prospective, comparative, observational study (Middle East: Lebanon, Kuwait, and the United Arab Emirates) | Metformin, Canagliflozin, Glibenclamide, Gliclazide, Glimepiride, Sitagliptin, Vildagliptin, Linagliptin & Saxagliptin | Fewer patients reported hypoglycemia in the SGLT2i group as compared to the Sulfonyl urea group. More patients treated with canagliflozin experienced volume depletion events compared with sulphonylurea. Both treatments were generally well tolerated, with low rates of adverse events and no serious adverse events in either group. |
| 27 | Efficacy and safety of liraglutide compared to sulphonylurea during Ramadan in patients with type 2 diabetes (LIRA-Ramadan): a randomized trial | T2DM | Open-label, active-controlled, parallel-group trial (Lebanon, Malaysia, UAE, Algeria, South Africa, India, Denmark & Israel) | Liraglutide 1.8 mg and sulphonylurea, both combined with metformin | Despite lower fructosamine levels and body weight at the beginning of Ramadan,  use of liraglutide showed similar glycaemic improvements, fewer hypoglycaemic episodes and greater body weight reduction compared with sulphonylurea. LIRA-Ramadan provides evidence for liraglutide being safe and efficacious for management of T2D during Ramadan fasting. |
| 28 | Effects of Exenatide (Exendin-4) on  Glycemic Control Over 30 Weeks in  Sulfonylurea-Treated Patients With Type  2 Diabetes | T2DM | Triple-blind, placebo-controlled, United States of America | Glimepiride,  Glipizide, Glyburide  Chlorpropamide &  Tolazamide | Exenatide at a fixed subcutaneous dose of 5 µg and 10 µg bid with long term usage can be a favorable option for the treatment of T2DM not adequately managed with sulfonyl ureas as it improved the overall glycemia (HbA1c) level. |
| 29 | A double-blind, randomized, multicenter study evaluating the effects of pioglitazone in fasting Muslim subjects during Ramadan | T2DM | Multicenter, double-blind randomized controlled trial, India | Pioglitazone, Metformin, acarbose | A remarkable improvement in glycemic control was observed when pioglitazone was used as an add on therapy with other OADs without any increase in Hypoglycemic event. However, significant weight gain was observed with the pioglitazone group. Unlike other class of drugs, dose adjustment is not required with pioglitazone. |
| 30 | Repaglinide plus single-dose insulin glargine: a safe regimen for low-risk type 2 diabetic patients who insist on fasting in Ramadan | T2DM | Prospective study, Turkey | Repaglinide plus single-dose insulin  glargine | None of the patients in fasting group reported a hypoglycemic event. The doses of  repaglinide and insulin glargine remained unchanged in both fasting and non-fasting groups throughout the study period. The participants reported neither a major nor a minor hypoglycemic event. The results indicated that this regimen was safe for low-risk type 2 diabetic patients who insisted on fasting during Ramadan. |
| 31 | Original paper: Efficacy and safety analysis of insulin degludec/insulin aspart compared with biphasic insulin aspart 30: A phase 3, multicentre, international, open-label, randomised, treat-to-target trial in patients with type 2 diabetes fasting during Ramadan | T2DM | Open label, Randomised trial (India, Lebanon, Malaysia, South Africa and Algeria) | Any basal, pre- or self-mixed insu  lin ± OADs for _90 days (OADs included metformin,  sulphonylureas, glinides, dipeptidyl peptidase-4 inhibitors, a-glucosidase inhibitors, sodium-glucose co-transporter-2  inhibitors) | In conclusion, compared with BIAsp 30 BID, IDegAsp BID had similar glycaemic control, which was maintained before, during and after Ramadan, despite the dose reductions at the beginning of Ramadan. IDegAsp BID was associated with significantly lower rates of overall and nocturnal symptomatic  hypoglycaemia during the 28-week treatment period and the 4-week Ramadan period. Despite significantly lower preiftar  SMPG values, IDegAsp was associated with a significantly lower rate of daytime hypoglycaemia compared with BIAsp  30. The results of this trial indicate the viability of IDegAsp BID as a therapeutic modality for patients on insulin who  choose to fast during Ramadan. |
| 32 | A comparison of insulin lispro Mix25TM and human insulin  30/70 in the treatment of type 2 diabetes during Ramadan | T2DM | Randomized open-label two period crossover comparative design (India, Pakistan, Malaysia, Singapore,  Egypt, Morocco, and South Africa) | Insulin lispro Mix25TM and Human insulin 30/70 | Though the number of hypoglycemic episodes was reported to be similar in both the groups, the treatment with lispro Mix25 was related with lesser average daily glycemia as compared to human insulin 30/70. There was no significant change reported in either of the groups with reference to body weight. |
