# Supplementary material for: Safety Assessment of Glucose-Lowering Drugs and Importance of Structured Education during Ramadan: A Systematic Review and Meta-Analysis
Source: J Diabetes Res. 2022 Feb 18;2022:3846253. doi: 10.1155/2022/3846253 (PMC8886741; doi:10.1155/2022/3846253)
Supplement: Supplementary 2 — Table 2: characteristics of classes of drugs and studies. [file 3846253.f2.docx]

**Table No 2: Author wise summary**

| **Sr. No** | **Author** | **Country** | **Type of Study** | **Sample size** | **Class of drugs** | **Drug involved** | **Outcome** |
| --- | --- | --- | --- | --- | --- | --- | --- |
| 01 | Bonakdaran S H & Khajeh-Dalouie | Iran | Observational | 17 | Biguanides and Sulfonyl ureas | Metformin,  Glibenclamide | Hyperglycemic and Hypoglycemic events. |
| 02 | A.H. Zargar et al | India, Pakistan & Bangladesh | Questionnaire | 136 | Sulfonylurea | Gliclazide | Fasting plasma glucose. |
| 03 | Al Arouj et al  (Virtue study) | Middle East & Asia | Prospective | 1333 | Biguanides, Sulfonylureas &  DPP4 inhibitors | Metformin, Vildagliptin,  Glimepiride,  Gliclazide,  Glibenclamide &  Glipizide | Hypoglycemic event,  Body weight,  HbA1c & overall safety assessed by adverse event (AE) and Serious AE monitoring. |
| 04 | Al Sifri et al | Middle East | Randomized trial | 1066 | Biguanides, Sulfonylureas &  DPP4 inhibitors | Metformin,  Sitagliptin,  Glimepiride,  Gliclazide &  Glibenclamide | **Primary endpoint:** Overall incidence of  symptomatic hypoglycemia  **Secondary endpoint:** Incidence of symptomatic or asymptomatic hypoglycemic events. |
| 06 | Anwar et al | Malaysia | Open label parallel group comparative trial | 41 | Biguanides,  Sulfonylurea &  Meglitinides | Metformin,  Repaglinide &  Glimepiride | Glycemic excursion, HbA1c & Hypoglycemic episodes. |
| 07 | GLIRA study group | Algeria, Egypt, Indonesia,  Jordan, Lebanon, and Malaysia | open-label, prospective,  observational study | 332 | Sulfonylurea | Glimepiride | HbA1c, fasting blood glucose & number of hypoglycemic events. |
| 08 | Cesur et al | Turkey | Open-label, multicenter, prospective, observational  study | 49 in fasting group & 16 non fasting group | Sulfonylurea, meglitinide and insulin | Glimepiride, repaglinide, and insulin glargine | Fasting blood  glucose (FBG), post-prandial blood glucose (PBG), HbA1c, and fructosamine as well as lipid metabolism. |
| 09 | Hassanein et al  (STEADFAST study) | Middle East, Europe, and Asia | Multiregional, double-blind study | 557 | Biguanides, Sulfonylurea & DPP4 inhibitors | Metformin,  Vildagliptin, Gliclazide,  Glibenclamide & Glipizide | Hypoglycemic events, HbA1c & weight change. |
| 10 | Al Arouj et al  (VIRTUE) | Middle East | Multicenter, prospective, observational cohort study | 584 | Biguanides, Sulfonylurea & DPP4 inhibitors | Metformin,  Vildagliptin, Glimepiride,  Gliclazide &  Glibenclamide | Hypoglycemic events, HbA1c, weight change, treatment exposure and adherence & adverse events. |
| 11 | Shete et al | India | Non-interventional, open-label, observational study | 97 | Biguanides, Sulfonylurea & DPP4 inhibitors | Glibenclamide, Gliclazide, Glimepiride &  Glipizide | Incidence  of hypoglycemic events (HEs), adverse events,  HbA1c, fasting  plasma glucose, postprandial plasma glucose and body  weight. |
| 12 | Malha et al | Lebanon | Randomized open-label clinical trial | 69 | Biguanides, Sulfonylurea & DPP4 inhibitors | Metformin,  Glimepiride/Gliclazide & Vildagliptin | HbA1c &  Hypoglycemia. |
| 13 | Halimi et al  (VERDI study) | France | Prospective, non-interventional study | 218 | Biguanides, Sulfonylurea & DPP4 inhibitors | Metformin &  Vildagliptin | **Primary assessment:** Incidence of all hypoglycemic episodes  **Secondary assessment:** Treatment adherence, HbA1c and body weight. |
| 14 | Seman et al | Malaysia | Randomized,  open-label, two-arm, parallel-group study | 119 | Biguanides, Sulfonylurea & SGLT2 inhibitor | Metformin, glimepiride,  gliclazide or glibenclamide &  Dapagliflozin | **Primary end point:** Hypoglycemia  **Secondary end point:** Adverse events, HbA1c, fasting  plasma glucose or fructosamine levels. |
| 15 | Hassanein et al  (CRATOS study) | Middle East | Non-randomised,  parallel-cohort,  prospective, comparative, observational study | 321 | Biguanides, Sulfonyl urea, DPP4 inhibitor and SGLT2 inhibitor | Metformin, Canagliflozin, Glibenclamide, Gliclazide, Glimepiride, Sitagliptin, Vildagliptin, Linagliptin & Saxagliptin | Hypoglycemic episodes, Volume depletion events, adverse events, HbA1c, BP, body weight, and eGFR. |
| 16 | Azar et al | Multi-site (7 countries) | Open-label, active-controlled, parallel-group trial | 343 | Biguanides, Sulfonyl urea & GLP-1 agonist | Metformin, Glibenclamide, Gliclazide, Glimepiride, Glipizide &  Liraglutide | Fructosamine, fasting plasma glucose, HbA1c, body weight, systolic (SBP) and diastolic (DBP) blood pressure. |
| 17 | Buse et al | USA | Triple-blind, placebo-controlled | 377 | Sulfonylurea &  GLP-1 agonist | Glimepiride,  Glipizide, Glyburide  Chlorpropamide &  Tolazamide | HbA1c, fasting plasma glucose, weight change, hypoglycemia and adverse events. |
| 18 | Vasan et al | India | Multicenter, double-blind randomized controlled trial | 76 | Thiazolidinedione, Biguanides,  Sulfonylurea, Meglitinides, α-glucosidase inhibitors | Pioglitazone, Metformin, acarbose | **Primary outcome:** Glycemic control assessed by serum fructosamine and number of hypoglycemic episodes  **Secondary outcome:** Evaluated the cost-effectiveness in using pioglitazone as an adjuvant form of therapy to conventional OHAs. |
| 19 | Bakiner et al | Turkey | Prospective study | 19 | Meglitinide &  Insulin | Repaglinide & Insulin glargine | Fasting blood glucose (FBG), Postprandial  blood glucose (PBG), Fructosamine level, body weight and blood pressure. |
